# Association between serum multi-protein biomarker profile and real-world disability in multiple sclerosis

**DOI:** 10.1093/braincomms/fcad300

**Published:** 2023-10-31

**Authors:** Wen Zhu, Chenyi Chen, Lili Zhang, Tammy Hoyt, Elizabeth Walker, Shruthi Venkatesh, Fujun Zhang, Ferhan Qureshi, John F Foley, Zongqi Xia

**Affiliations:** 1 Department of Neurology, University of Pittsburgh, Pittsburgh, PA, USA; 1 Department of Neurology, University of Pittsburgh, Pittsburgh, PA, USA; 1 Department of Neurology, University of Pittsburgh, Pittsburgh, PA, USA; Rocky Mountain Multiple Sclerosis Clinic, Salt Lake City, UT, USA; 1 Department of Neurology, University of Pittsburgh, Pittsburgh, PA, USA; 1 Department of Neurology, University of Pittsburgh, Pittsburgh, PA, USA; Octave Bioscience, Inc., Menlo Park, CA, USA; Octave Bioscience, Inc., Menlo Park, CA, USA; Rocky Mountain Multiple Sclerosis Clinic, Salt Lake City, UT, USA; 1 Department of Neurology, University of Pittsburgh, Pittsburgh, PA, USA

**Keywords:** multiple sclerosis, biomarkers, patient-reported outcome, disability, machine learning

## Abstract

Few studies examined blood biomarkers informative of patient-reported outcome (PRO) of disability in people with multiple sclerosis (MS). We examined the associations between serum multi-protein biomarker profiles and patient-reported MS disability. In this cross-sectional study (2017–2020), adults with diagnosis of MS (or precursors) from two independent clinic-based cohorts were divided into a training and test set. For predictors, we examined seven clinical factors (age at sample collection, sex, race/ethnicity, disease subtype, disease duration, disease-modifying therapy [DMT], and time interval between sample collection and closest PRO assessment) and 19 serum protein biomarkers potentially associated with MS disease activity endpoints identified from prior studies. We trained machine learning (ML) models (Least Absolute Shrinkage and Selection Operator regression [LASSO], Random Forest, Extreme Gradient Boosting, Support Vector Machines, stacking ensemble learning, and stacking classification) for predicting Patient Determined Disease Steps (PDDS) score as the primary endpoint and reported model performance using the held-out test set. The study included 431 participants (mean age 49 years, 81% women, 94% non-Hispanic White). For binary PDDS score, combined feature input of routine clinical factors and the 19 proteins consistently outperformed base models (comprising clinical features alone *or* clinical features plus one single protein at a time) in predicting severe (PDDS ≥ 4) versus mild/moderate (PDDS < 4) disability across multiple machine learning approaches, with LASSO achieving the best area under the curve (AUC_PDDS_ = 0.91) and other metrics. For ordinal PDDS score, LASSO model comprising combined clinical factors and 19 proteins as feature input (*R*^2^_PDDS_ = 0.31) again outperformed base models. The two best-performing LASSO models (*i.e.*, binary and ordinal PDDS score) shared six clinical features (age, sex, race/ethnicity, disease subtype, disease duration, DMT efficacy) and nine proteins (cluster of differentiation 6, CUB-domain-containing protein 1, contactin-2, interleukin-12 subunit-beta, neurofilament light chain [NfL], protogenin, serpin family A member 9, tumor necrosis factor superfamily member 13B, versican). By comparison, LASSO models with clinical features plus one single protein at a time as feature input did not select either NfL or glial fibrillary acidic protein (GFAP) as a final feature. Forcing either NfL or GFAP as a single protein feature into models did not improve performance beyond clinical features alone. Stacking classification model using five functional pathways to represent multiple proteins as meta-features implicated those involved in neuroaxonal integrity as significant contributors to predictive performance. Thus, serum multi-protein biomarker profiles improve the prediction of real-world MS disability status beyond clinical profile alone or clinical profile plus single protein biomarker, reaching clinically actionable performance.

## Teaser

Zhu *et al*. reported that serum multi-protein biomarker profiles improved the prediction of patient-reported disability status beyond clinical profile alone or clinical profile plus one single protein (such as neurofilament light chain or glial fibrillary acid protein) and may have clinical application in real-world monitoring of multiple sclerosis.

## Graphical Abstract

**Graphical abstract:**
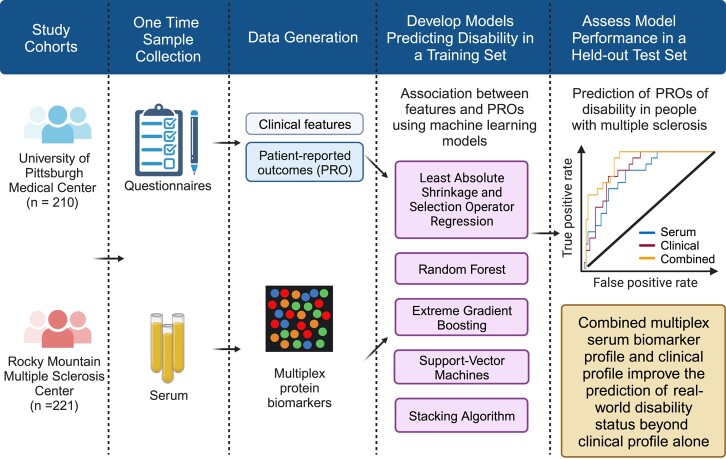

## Introduction

Multiple sclerosis (MS) is a chronic neurological disease that could cause progressive accumulation of neurological disability.^1,2^ People with MS (pwMS) exhibit individual variations in disease activity and progression trajectory.^3,4^ Existing MS disease-modifying therapies (DMTs) pose soaring costs and exhibit variable real-world effectiveness in preventing inflammatory disease activity and delaying disability worsening.^5-7^ In current practice, clinicians primarily rely on history, exams and neuroimaging to assess MS disease activity, disability progression and treatment response. There is an unmet need to improve disease monitoring at the point of care to guide individualized management.

Blood biomarkers could potentially aid MS monitoring.^8-11^ Serum neurofilament light chain (sNfL) and glial fibrillary acidic protein (sGFAP) are well-studied blood biomarkers of MS.^12-27^ NfL and GFAP are neuron-specific and astrocyte-derived intermediate filament cytoskeletal proteins, respectively.^12,18^ Blood NfL (e.g. sNfL) has potential clinical applications in monitoring neuroaxonal damage associated with inflammatory disease activity (i.e. clinical and/or radiological relapse), short-term disability worsening, and treatment response, though its utility to inform long-term disability remains unsettled. Blood GFAP (e.g. sGFAP) has potentially added utility in monitoring MS disability worsening, though it has limited utility to inform acute relapse. As real-world data such as patient-reported outcomes (PROs) gain importance in the clinical setting for MS disease monitoring, at least one previous study reported the correlation between baseline sNfL and PROs relevant to physical function.^28,29^ To our knowledge, no study has examined the association between blood multi-protein biomarker profile (beyond NfL or GFAP alone) and patient-reported MS disability.

Leveraging the Proximity Extension Assay (PEA) on the Olink™ platform,^30^ prior feasibility studies reported the analytical and clinical validation of a custom proteomic multiplex immunoassay (PMI) panel of multiple serum proteins, including sNfL and sGFAP, pertaining to key biological pathways in MS pathogenesis and associated with MS disease activity (i.e. the presence and count of gadolinium-enhanced lesions, annualized relapse rate) and other MS severity endpoints (*i.e.*, brain atrophy and rater-determined disability status).^31,32^ Here, we tested multiple statistical and machine learning (ML) approaches to examine the performance of biomarker profiles based on the same PMI panel of multiple serum proteins in predicting patient-reported disability status in pwMS. Specifically, we hypothesized that serum multi-protein profiles would improve the prediction of real-world disability status when compared to clinical profile or each single protein.

## Methods

### Study design and cohorts

**Figure 1:**
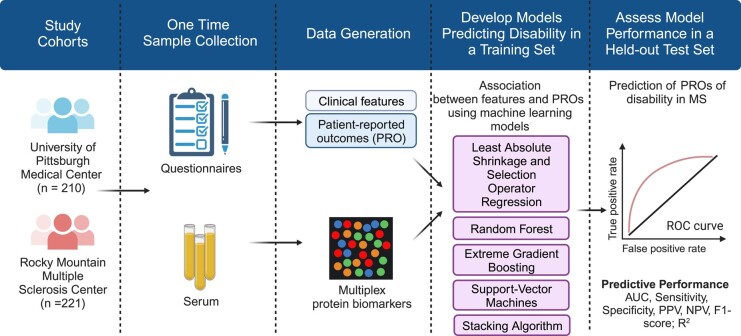
**Overall study design.** (Created with BioRender.com)

In this cross-sectional observational study (Fig. 1), we recruited participants from two independent MS centres in the USA: University of Pittsburgh Medical Center (UPMC, Pittsburgh, PA: *n* = 210) and Rocky Mountain Multiple Sclerosis Clinic (RMMSC, Salt Lake City, UT: *n* = 221) during 2017–2020. The study criteria included adults 18 years or older with a neurologist-confirmed diagnosis of MS according to the 2017 McDonald criteria,^14^ clinically isolated syndrome (CIS) or radiologically isolated syndrome. We did not exclude RIS (UPMC, *n* = 4; RMMSC, *n* = 0) or CIS (UPMC, *n* = 1; RMMSC, *n* = 0) given our goal to test in broad real-world clinic-based populations. We collected clinical and demographic data through review of electronic health records. Participants completed patient-reported outcomes (PROs) using either electronic or paper questionnaires. Participants donated venous blood samples during routine clinical appointments. Serum samples were isolated within 4 hours of phlebotomy and frozen at −80°C until proteomic profiling.

### Ethics approval

The institutional review boards of the University of Pittsburgh (STUDY19080007) and RMMSC (WCG20201562) approved the study protocols. All participants provided written informed consent.

### Serum protein biomarker profile

**Figure 2:**
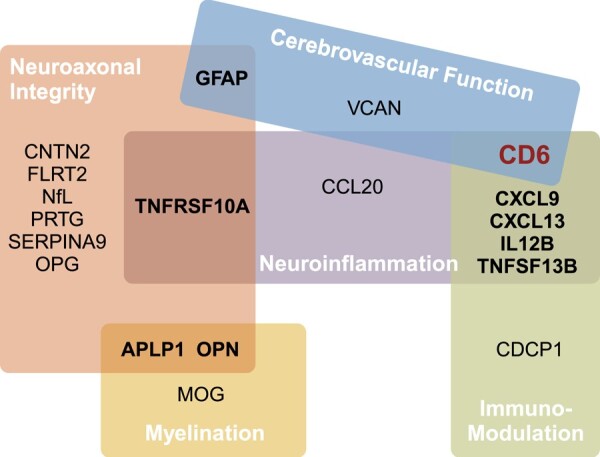
**Functional pathways.** The 19 protein biomarkers are grouped into five functional pathways: Cerebrovascular Function, Immunomodulation, Myelination, Neuroaxonal Integrity and Neuroinflammation. APLP1, amyloid beta precursor like protein 1; CCL20, chemokine (C-C motif) ligand 20; CD6, cluster of differentiation 6; CDCP1, CUB-domain-containing protein 1; CNTN2, contactin-2; CXCL9, chemokine (C-X-C motif) ligand 9; CXCL13, chemokine (C-X-C motif) ligand 13; FLRT2, fibronectin leucine-rich transmembrane protein 2; GFAP, glial fibrillary acidic protein; IL12B, interleukin-12 subunit beta; MOG, myelin oligodendrocyte glycoprotein; NfL, neurofilament light chain; OPG, osteoprotegerin; OPN, osteopontin; PRTG, protogenin; SERPINA9, serpin family A member 9; TNFSF10A, tumor necrosis factor ligand superfamily member 10; TNFSF13B, tumor necrosis factor ligand superfamily member 13B; VCAN, versican. (Created with BioRender.com)

Previously developed on the Olink™ platform using oligonucleotide-labelled antibodies and PEA methodology, a custom PMI panel comprising 19 proteins (see detailed protein names in Supplementary Table 1) measured the absolute concentration (pg/mL) of each serum protein.^31^ The key advantage of this multiplex approach is its potential clinical feasibility given its ability to assay multiple serum proteins in parallel using the same blood volume as for a single protein. In prior feasibility studies, a library of >1400 proteins was screened for association with standard MS disease activity endpoints, including clinically defined relapse versus remission, the presence (and count) versus absence of gadolinium-enhanced lesions on magnetic resonance imaging (MRI), annualized relapse rate, and Expanded Disability Status Scale (EDSS) score.^31,32^ The 19 proteins in the custom panel were selected based on optimal performance for predicting these MS clinical end points with a primary focus on inflammatory disease activity (i.e. clinical and/or radiological relapse). We further assigned the 19 proteins to five biological pathways relevant to MS pathogenesis (Cerebrovascular Function, Immunomodulation, Myelination, Neuroaxonal Integrity and Neuroinflammation) (Fig. 2).^33^ In this study, serum concentrations of the 19 proteins constituted the patient-level multi-protein biomarker profile.

Serum samples were assayed in five batches. No batch adjustment in subsequent analyses was necessary because principal component analyses showed no significant batch effects (Supplementary Fig. 1). We performed log transformation of protein concentrations to minimize outlier effects.

### Clinical profile

We collected standard clinical and demographic features that may influence MS disability: age (at sample collection), sex (female versus male), self-reported race/ethnicity (non-Hispanic white versus otherwise), disease subtypes (RIS/CIS/relapse-remitting MS versus progressive MS), disease duration (years between MS or precursor diagnosis and sample collection) and DMT efficacy at PRO assessment (high-efficacy versus standard-efficacy versus no DMT) as well as the time interval between serum collection and the closest PRO assessment (when they were not on the same day).^34-36^ The dichotomy of race/ethnicity subgroups was statistically necessary due to the modest number of participants who did not self-report as non-Hispanic white. For DMT in the study populations, we operationally categorized natalizumab, ocrelizumab and rituximab as higher-efficacy, while dimethyl fumarate, fingolimod, glatiramer acetate, interferon beta and teriflunomide as standard-efficacy.^7^ While certain DMT (e.g. fingolimod) could have been categorized as intermediate-efficacy, its modest sample size (total *n* = 6) did not warrant a separate DMT category.

### Feature sets

We created four input feature sets: (i) clinical profile only, (ii) clinical profile plus one single protein (out of 19) at a time, (iii) serum biomarker profile containing 19 proteins and (iv) combined clinical profile and serum multi-protein biomarker profile (Supplementary Table 2). We compared the feature set combining the clinical profile and serum multi-protein biomarker profile against other benchmark feature sets.

### Patient-reported outcomes of disability

We assessed real-world neurological and physical functions using two clinically relevant and interrelated PROs. First, the Patient Determined Disease Steps (PDDS) scale evaluates gait function, ranging from 0 to 8, with 0 indicating no gait impairment and 8 representing bed-bound status. PDDS complements rater-assessed EDSS.^37^ Second, the National Institute of Health Patient-Reported Outcomes Measurement Information System (PROMIS) Physical Function (version 1.2) quantifies general physical function. Nationally validated, PROMIS is a computer-adaptive test to measure patient-reported health across a range of chronic diseases and demographics, including MS.^38,39^ PROMIS reports a T-score and standard deviation (SD) relative to the general US population, which has a mean T-score of 50 (SD = 10). Higher PROMIS scores indicate better physical function or lower physical disability. While both are validated in pwMS, PDDS is MS-specific whereas PROMIS is a generalizable measure of real-world disability. Both study sites administered PDDS, while PROMIS data were only available from UPMC. We included PROs surveyed on or after the blood sample collection day for analysis. We used PROs as both ordinal/continuous and binary variables. We dichotomized PDDS according to the requirement for full-time ambulatory assistance (≥4 versus <4)^40^ and PROMIS based on the disability severity (≥35 mild/moderate disability versus <35 severe disability).^41,42^ We designated PDDS as the primary and PROMIS as the secondary outcome.

### Training and held-out test set

Findings based on diverse cohorts with different characteristics would only strengthen the study generalizability. Given the clinical and demographic differences between the two study cohorts (Table 1), we first split samples and data into 80:20 for a training and a held-out test set within each cohort. We then combined the training sets and held-out test sets from both cohorts into one training and one test set for subsequent analyses using PDDS. For the subgroup analyses using PROMIS, we used the 80:20 split from only the UPMC cohort.

**Table 1:** Patient characteristics

	UPMC (*n* = 210)	RMMSC (*n* = 221)	*P*-value
Age (years, mean ± SD)	48.4 ± 12.3	49.1 ± 12.4	0.59
Sex (*n*, % of cohort)			0.65
Female	172 (81.9)	177 (80.0)	
Male	38 (18.1)	44 (20.0)	
Race (% of cohort)			0.002
White	192 (91.4)	216 (97.7)	
Black/African American	15 (7.1)	1 (0.5)	
Asian	2 (1.0)	1 (0.5)	
Not reported	1 (0.5)	3 (1.4)	
Ethnicity (% of cohort)			0.04
Not Hispanic	207 (98.6)	208 (94.1)	
Hispanic	2 (1.0)	11 (5.0)	
Not reported	1 (0.5)	2 (0.9)	
Disease duration (years, mean ± SD)	12.0 ± 9.8	13.8 ± 9.8	0.06
Disease subtype (*n*, % of cohort)			<0.001
RRMS and precursors (RIS, CIS)	196 (93.3)	217 (98.2)	
PMS	14 (6.7)	4 (1.8)	
DMT efficacy^a^ (*n*, % of cohort)			<0.001
No DMT	38 (18.1)	9 (4.1)	
Standard efficacy	80 (38.1)	40 (18.1)	
Higher efficacy	92 (43.8)	172 (77.8)	
Active relapse (*n*, % of cohort)	1 (0.5)^b^	5 (2.3) ^c^	0.11
PDDS (median, IQR)	1 (3)	1 (3)	0.23
PDDS < 4 (*n*, % of cohort)	166 (75.9)	185 (83.7)	0.11
PDDS time^d^ (days, mean ± SD)	73.4 ± 97.9	0.0 ± 0.0	<0.001
PROMIS (mean, SD)	43.8 ± 10.3		
PROMIS < 35 (*n*, % of cohort)	37 (20.1)		
PROMIS time^e^ (days, mean ± SD)	350.2 ± 303.6		

^a^DMT efficacy was coded as 0 = None, 1 = standard efficacy, 2 = higher efficacy at the time of PRO assessment. In the study dataset, natalizumab, rituximab and ocrelizumab were operationally categorized as high-efficacy therapies while dimethyl fumarate, fingolimod, glatiramer acetate, interferon beta and teriflunomide were categorized as standard efficacy.

^b^Active relapse in the UPMC cohort was operationally defined as clinical and/or radiological relapse within 30 days prior to sample collection.

^c^Active relapse in the RMMSC cohort was operationally defined as infusion of methylprednisolone for treatment of acute relapse in the 30 days prior to sample collection.

^d^PDDS time was defined as the time interval between serum collection and the closest PDDS assessment after sample collection. All RMMSC samples were collected on the same day as the PDDS assessment, while UPMC samples were collected either before or on the same day as the PDDS assessment.

^e^PROMIS time was defined as the time interval between serum collection and the closest PROMIS assessment after sample collection. The RMMSC cohort did not collect PROMIS.

Abbreviations: SD = standard deviation; IQR = interquartile range; RRMS = relapse-remitting MS; PMS = (primary or secondary) progressive MS; CIS = clinical isolated syndrome; RIS = radiological isolated syndrome; DMT = disease-modifying therapies; PDDS = Patient Determined Disease Steps; PROMIS = Patient-Reported Outcomes Measurement Information System, physical function.

### Machine learning methods

**Figure 3:**
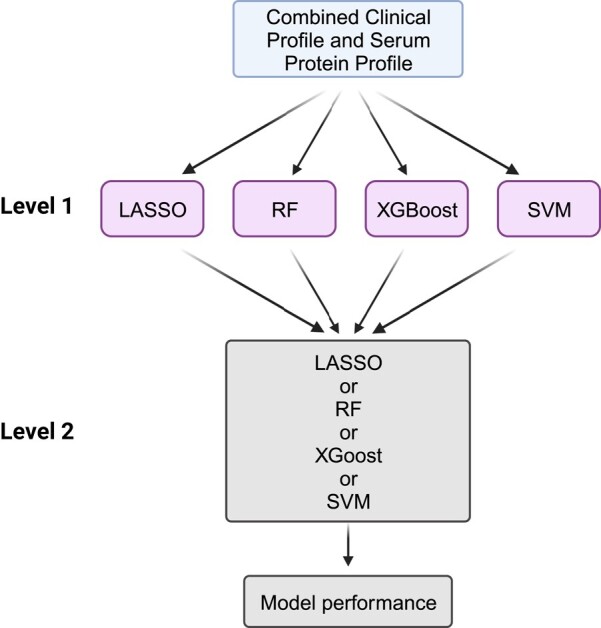
***Stacking ensemble* learning.** The Level 1 models include four different machine learning methods (LASSO [Least Absolute Shrinkage and Selection Operator], RF [Random Forest], XGBoost [Extreme Gradient Boosting] and SVM [Support Vector Machine]) as ensemble members. The Level 2 model then utilizes the predictions of the Level 1 models to perform the outcome prediction. (Created with BioRender.com)

**Figure 4:**
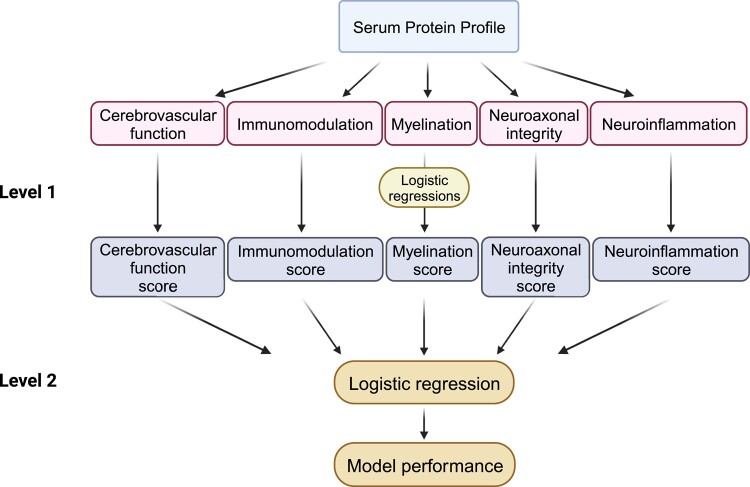
***Stacking classification* algorithm using functional pathways as meta-features in the predictive models.** The Level 1 logistic regression models use the biomarker concentrations as raw feature inputs to generate coefficients for biomarkers in each pathway and produce a probability score for the pathway. The Level 2 logistic regression uses the predicted probability scores of the functional pathways as meta-feature input. (Created with BioRender.com)

As different ML approaches have known strengths and weaknesses, we systematically deployed the following ML approaches to test the best prediction of PROs (Supplementary Table 2): (i) Least Absolute Shrinkage and Selection Operator (LASSO) regression, (ii) Random Forest (RF), (iii) Extreme Gradient Boosting (XGBoost), (iv) Support Vector Machines (SVM), (v) stacking ensemble learning and (vi) stacking classification algorithm. LASSO performs penalized L1 regularization, which produces sparse models containing the minimal number of final informative features.^43^ RF creates a collection of random uncorrelated decision trees to produce the best possible prediction.^44^ XGBoost optimally combines decision tree and linear regression under a Gradient Boosting framework, which efficiently decreases errors and effectively reduces irrelevant features.^45^ SVM performs supervised classifications to map features into discrete spaces to maximize the gap between data points in separate categories.^46^ Stacking ensemble learning combines the best predictions from two or more base ML methods (Fig. 3).^47^ Finally, stacking classification algorithm, which differs from stacking ensemble learning, enables proteins organized in functional pathways as feature input in predictive models in two steps: (i) The first-level logistic regression models use biomarker concentrations as raw feature inputs to generate coefficients for the biomarkers in each given pathway (y = β_0_ + β_1_×biomaker_1_ + … + β*_n_* × biomaker_n_) and produce a probability score ($p=\;\frac{1}{1+\;e^{y}}$) for the pathway; (ii) The second-level logistic regression uses the predicted probability scores of the functional pathways as meta-features for model input (Fig. 4).^48^

To report model performance, we assessed the area under the receiver operating characteristic curve (AUC) with 95% confidence interval (CI) computed with 2000 bootstrap replicates as well as sensitivity, specificity, positive predictive value (PPV) or precision, negative predictive value (NPV), and F1-score for *binary outcomes* as well as variance explained (*R*^2^) with 95% confidence intervals (95% CI) for *continuous/ordinal outcomes*. We assessed 95% CIs, and *P*-values for AUC comparisons of all models to the model with combined clinical and serum multi-protein profiles non-parametrically by bootstrapping with 1000 replicates.

### Additional statistical analysis

We compared cohort characteristics using chi-square test for categorical data and *t*-test for continuous variables. We performed the Spearman correlation across all variables. For these tests, *P*-value <0.05 was deemed statistically significant. We performed all analyses using R, version 4.0.2.

### Data availability

Code for analysis and figures is available at < https://github.com/xialab2016/MSbiomarker.git > . De-identified data are available upon request to the corresponding author and with permission from the participating institutions.

## Results

### Patient characteristics

This study included 431 participants with MS (or precursor) diagnosis (UPMC: *n* = 210; RMMSC: *n* = 221; Table 1). The two cohorts shared similar age, sex and disease duration as well as the proportion with active relapse within 30 days prior to sample collection and the proportion with mild/moderate disability (PDDS < 4). When compared to RMMSC, the UPMC cohort had a lower percentage of White (91.4% versus 97.7%, *P* = 0.002), Hispanic (1.0% versus 5.0%, *P* = 0.04) patients, higher percentage with progressive MS (6.7% versus 1.8%, *P* < 0.001) and receiving no DMT (18.1% versus 4.1%, *P* < 0.001) or standard-efficacy DMT (37.5% versus 18.1%, *P* < 0.001) at sample collection. While all RMMSC participants completed PDDS assessment on the same day as serum sample collection, the time interval (mean ± SD) between sample collection and the closest PDDS after sample collection was 75.5 ± 98.4 days for UPMC participants. UPMC participants additionally completed PROMIS assessment, with a time interval of 350.2 ± 303.6 days between sample collection and the closest PROMIS after sample collection.

### Feature correlation

To assess the correlation structure of the available input features for predictive models, we assessed their pairwise correlations (Supplementary Fig. 2). Among clinical features, disease duration and age showed the strongest correlation (*r* = 0.57, *P* < 0.001). Among serum proteins, most significant correlations were positive, with myelin oligodendrocyte glycoprotein (MOG) and amyloid beta precursor like protein 1 (APLP1) concentrations having the strongest correlation (*r* = 0.67, *P* < 0.0001), while only GFAP and CD6 displayed inverse correlation (*r* = −0.39, *P* < 0.0001). When assessing serum proteins with clinical features, NfL concentrations and age showed the strongest positive correlation (*r* = 0.56, *P* < 0.001), whereas CXCL13 and DMT efficacy had the strongest inverse correlation (*r* = −0.26, *P* < 0.001).

### Best ML model performance for predicting PRO of disability

**Figure 5:**
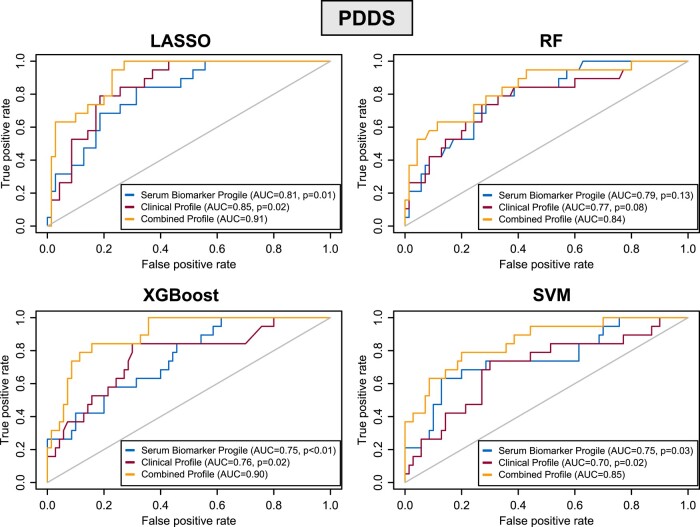
**Receiver operating characteristic (ROC) plots.** For predicting severe versus mild/moderate patient-reported MS disability using Patient Determined Disease Steps (PDDS ≥ 4 versus < 4), we systematically tested multiple machine learning models (LASSO [least absolute shrinkage and selection operator], RF [random forest], XGBoost [Extreme Gradient Boosting] and SVM [support vector machine]). We assessed 95% CIs and *P*-values for AUC comparisons of all models to the best-performing model non-parametrically by bootstrapping with 1000 replicates. *P*-values indicate the statistical significance when compared to the best-performing model using the combined feature input comprising clinical profile plus serum multi-protein biomarker profile. D-values quantify the difference in performance (AUC) between the two models while accounting for the variability (standard error) of the difference, with a higher D-value indicating a greater difference in performance between the models. LASSO: clinical profile (*P* = 0.02, D = 2.053), serum biomarker profile (*P* = 0.01, D = 2.492); RF: clinical profile (*P* = 0.08, D = 1.383), serum biomarker profile (*P* = 0.13, D = 1.147); XGBoost: clinical profile (*P* = 0.02, D = 2.152), serum biomarker profile (*P* < 0.01, D = 2.678); SVM: clinical profile (*P* = 0.02, D = 2.023), serum biomarker profile (*P* = 0.03, D = 1.948).

Overall, the LASSO approach consistently outperformed alternative ML approaches for predicting the primary endpoint, PDDS score, a MS-specific PRO of disability (Fig. 5, Table 2). Further, the combined clinical profile and serum multi-protein biomarker profile (containing 19 proteins) as feature input consistently outperformed benchmark feature sets.

**Table 2:** LASSO model performance and coefficients of the final feature set for predicting patient determined disease steps (PDDS) score

	PDDS binary			PDDS ordinal		
	Clinical features only	Multi-proteins only	Clinical features + multi-proteins	Clinical features only	Multi-proteins only	Clinical features + multi-proteins
AUC (95% CI)	0.85 (0.77–0.93)	0.81 (0.71–0.91)	0.91 (0.85–0.97)			
Sensitivity	0.60	0.71	0.89			
Specificity	0.81	0.83	0.86			
PPV	0.16	0.26	0.42			
NPV	0.97	0.97	0.99			
F1-score	0.25	0.38	0.57			
*R*2 (95% CI)				0.20 (0.07–0.33)	0.28 (0.16–0.40)	0.31 (0.20–0.41)
	**Clinical features** ^a^					
Age	0.00944		0.00824	0.03861		0.02797
Sex	−0.07995		−0.05932	−0.30695		−0.22614
Race/ethnicity	0.08149		0.06346	0.22352		0.14558
Disease subtype	0.00001		0.00001	0.00006		0.00006
Disease duration	0.04749		0.00880	0.23536		0.08613
DMT efficacy	0.05007		0.02673	0.14893		0.05732
PDDS time	0.00033		0.00016	NS		NS
	**Protein features** ^b^					
APLP1		NS	NS		−0.30884	−0.14282
CCL20		NS	NS		NS	NS
CD6		−0.09263	−0.06976		−0.16004	−0.12296
CDCP1		0.09062	0.02342		0.69860	0.46535
CNTN2		0.09778	0.05986		0.27394	0.00893
CXCL13		NS	NS		NS	NS
CXCL9		0.09633	0.04795		0.18378	NS
FLRT2		NS	NS		NS	NS
GFAP		0.00350	NS		0.18602	0.12661
IL12B		−0.07726	−0.04695		−0.43279	−0.26344
MOG		−0.08281	−0.09053		NS	NS
NfL		0.09751	0.00694		0.45991	0.08793
OPG		NS	0.00385		NS	NS
OPN		NS	NS		NS	NS
PRTG		−0.11618	−0.05996		−0.72771	−0.42898
SERPINA9		−0.00412	−0.00443		−0.06799	−0.05539
TNFRSF10A		−0.00070	NS		NS	NS
TNFSF13B	-	0.10373	0.07722	-	0.48581	0.32122
VCAN	-	−0.16151	−0.10457	-	−0.77226	−0.56348

^a^Please refer to Table 1 and its footnotes for detailed explanation of the clinical features.

^b^Please refer to Fig. 2 and Supplementary Table 1 for the full names of the protein biomarkers.

Abbreviations: AUC = area under the receiver operating characteristics curve; 95% CI = 95% confidence interval; PPV = positive predictive value; NPV = negative predictive value; PDDS = Patient Determined Disease Steps; NS = not selected by LASSO due to zero coefficient.

We assessed the model performance in the held-out test set for predicting PDDS score as either a binary or ordinal measure. When predicting severe versus mild/moderate MS disability (binary PDDS ≥ 4 versus < 4), the LASSO model using the combined clinical profile plus serum multi-protein biomarker profile as feature input achieved the best AUC (0.91, 95% CI 0.85–0.97) when compared to benchmark feature sets [clinical profile alone (AUC 0.85, 95% CI 0.77–0.93, *P* = 0.02), serum multi-protein biomarker profile alone (AUC 0.81, 95% CI 0.71–0.91, *P* = 0.01)] (Table 2). Beyond AUC, the LASSO model with the combined feature set attained better sensitivity (0.89), specificity (0.86), PPV (0.42), NPV (0.99) and F1-score (0.57) than LASSO models with benchmark feature sets. To put this in a clinical context, the LASSO model with the combined feature set would correctly predict 26 more patients with severe disability per 100 MS patients tested, when compared to the LASSO model using clinical profile only. Likewise, when predicting PDDS as ordinal score, the LASSO model with the combined feature set again achieved the best variance explained *R*^2^ (0.31, 95% CI 0.20–0.41) (Table 2).

The two best overall performing LASSO models (both with the combined feature set) for predicting PDDS score (as either binary or ordinal score) selected slightly different final sets of informative features (Table 2). Collectively, these two models shared the following final sets of features, each showing the same direction of association between them: 6 out of 7 clinical features (age, sex, race/ethnicity, disease subtype, disease duration, DMT efficacy) and 9 out of 19 proteins (CD6, CDCP1, CNTN2, IL12B, NfL, PRTG, SERPINA9, TNFSF13B, VCAN) (Table 2, Supplementary Fig. 4).

To further explore the contribution of each serum protein in model performance, we systematically assessed LASSO models using clinical profile plus *individual* protein (i.e. one single protein at a time rather than the multi-protein biomarker profile) as feature input for predicting severe disability (binary PDDS ≥ 4 versus < 4). Adding a single protein as an input feature did not significantly improve LASSO model performance beyond the clinical profile alone (AUC 0.85, 95% CI 0.77–0.93) (Supplementary Table 3). Notably, LASSO models using clinical profile plus single protein as feature input did *not* select five proteins as the final features, including the well-known NfL and GFAP. When forcing either NfL or GFAP (as a single protein) into the respective LASSO models, none of the model performance metrics improved when compared to clinical profile alone (Supplementary Table 3).

In subgroup analyses predicting the secondary endpoint, PROMIS-physical function, a generalizable PRO of physical disability (UPMC cohort only, *n* = 210), the LASSO approach also overall outperformed alternative ML approaches (Supplementary material, Supplementary Fig. 3, Supplementary Table 4). Again, the combined clinical profile plus serum multi-protein biomarker profile as feature input largely outperformed benchmark feature sets.

### Model performance of alternative ML approaches and the ensemble method

Like LASSO models, RF, XGBoost or SVM models with the combined feature set as input overall outperformed benchmark feature sets (Supplementary Table 5). When comparing the alternative ML approaches against LASSO (all with the combined feature set) for predicting severe MS disability (using binary PDDS score), the alternative ML approaches (RF, XGBoost, SVM) underperformed in most performance metrics (Fig. 5, Table 2). Specifically, RF achieved better specificity and PPV but at the cost of markedly worse sensitivity as well as worse AUC, NPV and F1-score. XGBoost achieved similar AUC but had markedly worse sensitivity as well as worse NPV and F1-score. SVM achieved similar NPV but showed worse AUC, PPV and F1-score as well as marginally worse sensitivity and specificity. In subgroup analyses (using binary PROMIS-physical function score), the alternative ML approaches also underperformed LASSO in predicting severe general physical disability across nearly all metrics (Supplementary Fig. 3, Supplementary Table 5).

We further tested whether a *stacking ensemble* approach using the combined feature set could further improve the predictive performance over the individual ML models (specifically over LASSO, but also RF, XGBoost or SVM). The stacking ensemble approach did not perform better than the best individual ML approach (i.e.LASSO) for predicting MS-specific or general disability (binary PDDS or PROMIS score: Supplementary Table 6). The lack of significant improvement could be due to the already robust performance of the best-performing LASSO model.

### Model performance using functional pathways

Given that the 19 proteins in the serum protein profile can be organized into five functional pathways, we examined the predictive performance of the five functional pathways as meta-feature input in predicting severe MS disability (binary PDDS score) in the held-out test set. In this analysis to corroborate the best-performing LASSO model, *stacking classification* model using clinical profile plus five functional pathways (to represent multi-protein profile) showed better overall model performance (AUC 0.86, 95% CI 0.74–0.86; sensitivity 0.95, specificity 0.62, PPV 0.44, NPV 0.98, F1-score 0.61) than the model using five functional pathways alone (AUC 0.77, 95% CI 0.65–0.77; sensitivity 0.81; specificity 0.61, PPV 0.40, NPV 0.91, F1-score, 0.53) (Table 3) or the model using clinical features alone (Table 2). When compared with the best-performing LASSO model using combined clinical and multi-protein profiles (AUC 0.91, 95% CI 0.85–0.97; sensitivity 0.89, specificity 0.86, PPV 0.42, NPV 0.99, F1-score 0.57), the stacking classification algorithm using combined clinical profile and five functional pathways as feature input achieved worse AUC and specificity (and marginally worse NPV) but better sensitivity, PPV and F1-score (Tables 2 and 3).

**Table 3:** Model performance of the stacking classification model and the significant functional pathways for predicting binary patient determined disease steps (PDDS) scores (≥4 versus < 4)

	Functional pathways	Clinical profile + functional pathways
AUC (95% CI)	0.77 (0.65–0.77)	0.86 (0.74–0.86)
Sensitivity	0.81	0.95
Specificity	0.61	0.62
PPV	0.40	0.44
NPV	0.91	0.98
F1-score	0.53	0.61

	Coefficient	*P*-value	Coefficient	*P*-value
	**Clinical features** ^a^			
Age			0.040	0.034
Sex			−0.474	0.254
Race/ethnicity			−0.474	0.254
Disease subtype			0.689	0.051
Disease duration			0.039	0.026
DMT efficacy			0.404	0.138
PDDS time			0.005	0.004
	**Functional pathways** ^b^			
Neuroaxonal integrity	0.903	<0.001	0.653	0.006
Immunomodulation	0.798	0.024	0.459	0.251
Myelination	−0.996	0.392	−1.566	0.218
Neuroinflammation	0.225	0.595	0.304	0.521
Cerebrovascular function	1.256	0.766	1.157	0.199

^a^Please refer to Methods and Table 1 for description of the clinical features.

^b^Please refer to Fig. 2 and Supplementary Table 1 for the proteins of each functional pathway.

Abbreviations: AUC = area under the receiver operating characteristics curve; 95% CI = 95% confidence interval; PPV = positive predictive value; NPV = negative predictive value.

The immunomodulation (*P* = 0.02) and the neuroaxonal integrity (*P* < 0.001) pathways significantly contributed to the predictive performance of the stacking classification algorithm using five functional pathways alone, while the neuroaxonal integrity (*P* = 0.006) pathway remained significant in the model comprising the combined clinical profile and the five functional pathways. Interestingly, the four proteins selected as final informative features by the four best-performing LASSO models using combined clinical and multi-protein profiles for predicting binary and ordinal/continuous PDDS and PROMIS scores (Table 2, Supplementary Table 4, Supplementary Fig. 4) are involved in either the neuroaxonal integrity pathway (NfL and PRTG) or the immunomodulation pathway (CDCP1 and IL-12B) (Fig. 2). The neuroaxonal integrity pathway also includes four proteins (APLP1, CNTN2, SERPINA9, TNFSF13B) shared by at least two or more best-performing LASSO models in predicting PDDS or PROMIS scores (Supplementary Fig. 4).

## Discussion

The key study finding is that the addition of serum biomarker profiles comprising multiple proteins primarily associated with MS inflammatory disease activity endpoints improved the model performance of machine learning approaches in predicting real-world disability status beyond clinical profile alone, reaching clinically actionable accuracy as well as other performance metrics. LASSO outperformed alternative machine learning approaches, including RF, XGBoost, SVM or stacking ensemble. Importantly, serum multi-protein biomarker profiles consistently outperformed single protein such as NfL or GFAP as model feature input. Proteins involved in neuroaxonal integrity significantly contributed to the predictive performance of serum multi-protein biomarker profile in conjunction with clinical profile.

Our study has several strengths. First, this is the first study to our knowledge that demonstrates the potential clinical application of serum *multi-protein* biomarker profile in predicting real-world MS disability status. Prior studies showed that individual blood protein biomarker such as sNfL or sGFAP alone was insufficient to accurately predict MS disability (or treatment response).^14,16,18,49^ In this study, the combined input feature set comprising standard clinical profile and serum multi-protein profile consistently outperformed not only clinical profile alone but importantly also clinical profile plus single protein biomarker at a time, including notably sNfL or sGFAP, in predicting patient-reported disability status in pwMS. A key clinical advantage of the multiplex assay used in this study is the ability to profile multi-protein biomarkers using similar blood volume as for measuring a single protein. Second, we employed a primary and a secondary PRO to represent real-world evidence of disability status. Both PDDS and PROMIS are well validated PROs in pwMS.^37-39^ In particular, PDDS correlates with and complements rater-determined Expanded Disability Status Scale,^37^ which is a standard measure of disability in MS clinical trials but has limited application in the real-world clinical settings. On the other hand, PROs such as PDDS and PROMIS are already widely used in routine clinical settings. Given that few prior studies examined the clinical utility of blood biomarkers in predicting PROs in MS and none focused on multi-protein profile and patient-reported disability status, this study contributes real-world evidence. Third, we systematically tested multiple machine learning approaches (each with strengths and weaknesses) that *consistently* confirmed the added utility of serum multi-protein biomarker profiles as feature input in robustly predicting real-world MS disability beyond the benchmark clinical profile alone or clinical profile plus single protein at a time such as NfL or GFAP. Fourth, stepwise accumulation of neurological damage due to incomplete recovery from acute relapse is a major contributor of disability in relapse-remitting MS. Our study demonstrated that the serum biomarker panel comprising multiple proteins developed based on their associations with primarily inflammatory MS disease activity endpoints in prior feasibility studies is crucially also informative in predicting real-world patient-reported MS disability status. Finally, we leveraged data from two independent cohorts with clinically and demographically distinct characteristics. By first splitting data into 80:20 for a training and a held-out test set within each cohort and then reporting the machine learning model performance in the combined held-out test set, the study findings have potentially greater generalizability than using a single cohort. Taken together, these findings suggest that serum biomarker profiles comprising multiple proteins better capture the complex disease states of pwMS (i.e. disability status) and may have clinical application in real-world monitoring of MS.

The protein biomarkers (CDCP1, IL-12B, NfL and PRTG) selected by the four best-performing LASSO models (for predicting the primary endpoint of PDDS and the secondary endpoint of PROMIS, both as binary and ordinal/continuous scores) are involved in the two known functional pathways (*i.e.,* immunomodulation, neuroaxonal integrity) pertinent to MS pathogenesis (Fig. 2, Supplementary Fig. 4). Notably, NfL and PRTG in the neuroaxonal integrity pathway significantly contributed to the performance of the stacking classification model using clinical profile and the five functional pathways as meta-features representing multi-protein profile. Further, four other proteins (APLP1, CNTN2, GFAP and SERPINA9) selected by two or more best-performing LASSO models (for predicting PDDS or PROMIS scores) are also involved in the same neuroaxonal integrity pathway. In a prior feasibility study, these four proteins were associated with MS disease activity.^50^ Further, the three proteins (APLP1, CNTN2, GFAP) in addition to the best known NfL play roles in MS pathogenesis, including demyelination and remyelination,^51,52^ grey matter pathology,^53^ and T cell dysregulation.^54,55^ Given the complex pathogenesis of MS, our findings suggested that a serum multi-protein biomarker profile encompassing proteins involved in different MS pathogenesis pathways is superior to a single protein biomarker in informing real-world MS disability status.

Our study has limitations. First, the current cross-sectional study design does not allow testing predictions of disability progression. While long-term follow-up study is under way, this study establishes important baseline findings. Second, disability status based on PROs might contain ascertainment or other biases. On the other hand, both PDDS and PROMIS scores are well-validated PROs against rater-assessed exams and crucially provide real-world evidence of neurological function much more readily attainable than EDSS.^37-39^ Third, the racial and ethnic composition of the two study cohorts limited the generalizability beyond the mostly non-Hispanic white population. The current study lays the conceptual framework for future testing in more demographically diverse populations.

## Conclusion

Serum multi-protein biomarker profiles based on proteomic multiplex immunoassay improve the prediction of real-world MS disability status beyond clinical profile alone or clinical profile plus single protein biomarker (e.g. NfL or GFAP), reaching clinically actionable performance. Future studies that include long-term clinical follow-up, incorporate objective functional testing in conjunction with PROs, and recruit higher proportions of participants from more diverse racial and ethnic backgrounds would further establish the clinical utility of this integrated approach in monitoring individual MS disease trajectories, including the prediction of relapse-free disability progression. With further validation, we will establish an open-source interface for exploring real-world clinical application while studying the feasibility of incorporating multi-protein biomarkers in clinical decision support.

## Supplementary Material

fcad300_Supplementary_Data
