# Supplementary material for: Association between serum multi-protein biomarker profile and real-world disability in multiple sclerosis
Source: Brain Commun. 2023 Oct 31;6(1):fcad300. doi: 10.1093/braincomms/fcad300 (PMC10773609; doi:10.1093/braincomms/fcad300)
Supplement: fcad300_Supplementary_Data [file fcad300_supplementary_data.pdf]

ONLINE-ONLY SUPPLEMENTS

Association between Serum Multi-Protein Biomarker Profile and Real-World Disability in Multiple Sclerosis

Supplementary Results... 2
Supplementary Figure 1... 3
Supplementary Figure 2... 4
Supplementary Figure 3... 5
Supplementary Figure 4... 6
Supplementary Table 1... 7
Supplementary Table 2... 8
Supplementary Table 3... 9-10
Supplementary Table 4... 11-12
Supplementary Table 5... 13
Supplementary Table 6... 14

SUPPLEMENTARY RESULTS

Model Performance for Predicting PRO of General Physical Function (Secondary End Point)

In subgroup analyses (UPMC cohort, n=210), the LASSO approach outperformed the other machine learning approaches (RF, XGBoost, SVM) in predicting the secondary endpoint of PROMIS-physical function, which is a PRO of general physical function (Supplementary Figure 3, Supplementary Table 4). The combined clinical profile and serum multi-protein biomarker profile (comprising 19 proteins) as feature input largely outperformed other benchmark feature sets.

We assessed model performance in the held-out test set for predicting PROMIS-physical function as either a binary or continuous score. Again, LASSO models using the combined clinical profile and serum multi-protein biomarker profile as feature input showed better performance (severe versus mild/moderate general physical disability, PROMIS <35 vs. ≥35: AUC=0.90, 95%CI 0.78-1.00; continuous PROMIS: R^2=0.35, 95%CI 0.29-0.42) than other benchmark feature sets or other ML models (Supplementary Figure 3, Supplementary Table 4, Supplementary Table 5). The subgroup analysis is likely underpowered to detect a statistically significant difference in performance between the LASSO model with combined feature set versus benchmark clinical profile alone in predicting binary PROMIS score. The two LASSO models (both with the combined feature set) for predicting PROMIS (as binary or continuous score) selected slightly different final sets of informative features, and collectively shared 2 clinical features (race/ethnicity, disease subtype) and 6 proteins (APLP1, CDCP1, GFAP, IL12B, NfL, PRTG) (Supplementary Figure 4, Supplementary Table 4).

When assessing the overlap of the features selected by the four LASSO models (with the combined feature set for predicting the primary endpoint of PDDS score and the secondary endpoint of PROMIS physical function score, each as binary or ordinal/continuous), 2 clinical features (race/ethnicity, disease subtype) and 4 proteins (CDCP1, IL-12B, NfL, PRTG) persisted (Supplementary Figure 4).

SUPPLEMENTARY FIGURES AND TABLES

Supplementary Figure 1. Principal component analysis of serum sampling showed no significant batch effects. The scatter plot demonstrates the first two principal components (PC, PC1 vs PC2) of each batch. ANOVA analyses show no significant difference of PC1 (F=1.28 x 10–29, p=1.00) and PC2 (F=1.80 x 10-29, p=1.00) across the batches.

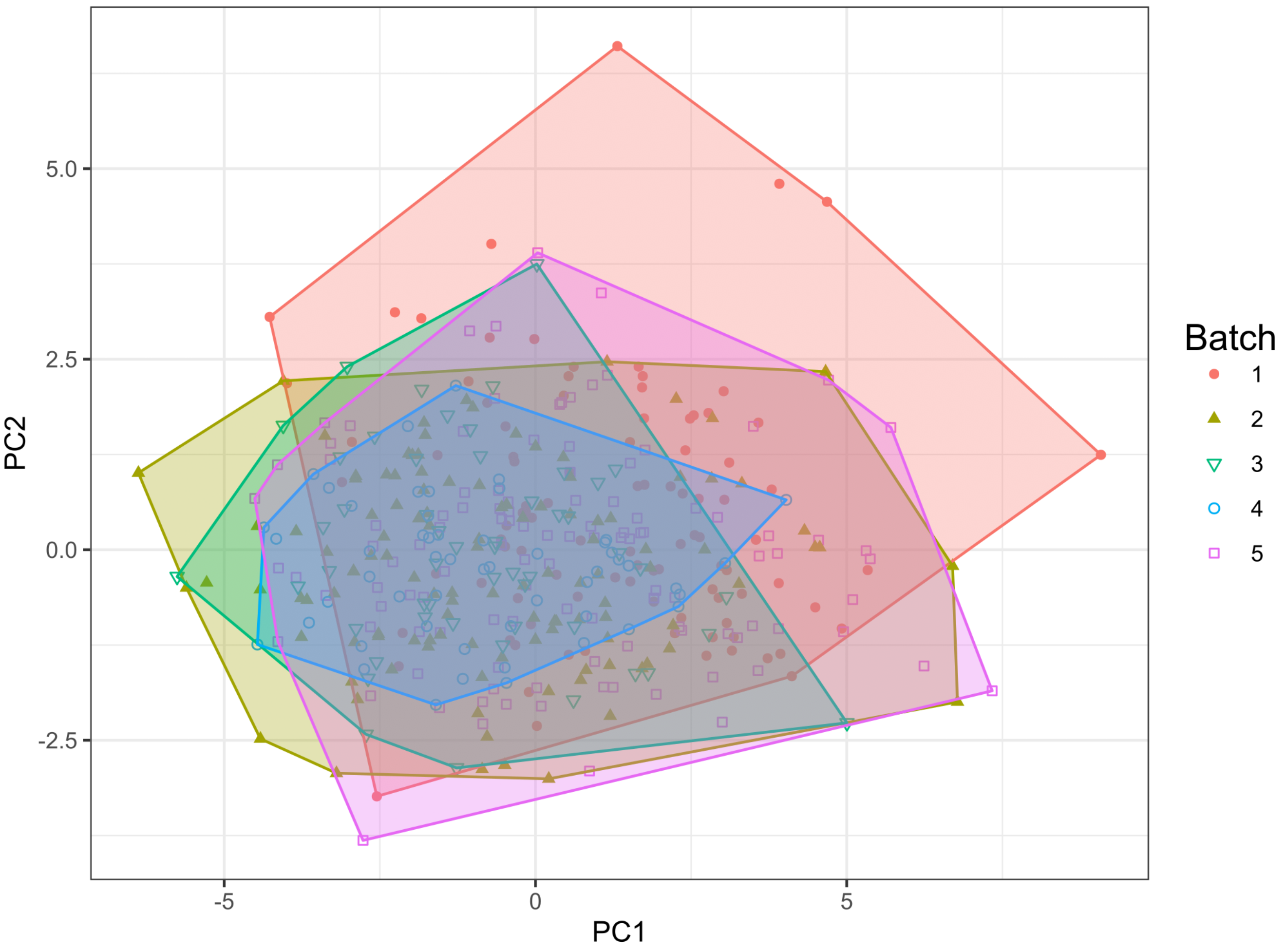

Supplementary Figure 2. Correlation structure of features.
Each square shows the pairwise Spearman correlation coefficient between pairs of features. The presence of the color indicates nominal significance (p<.05) with blue indicating positive and red indicating inverse correlation.

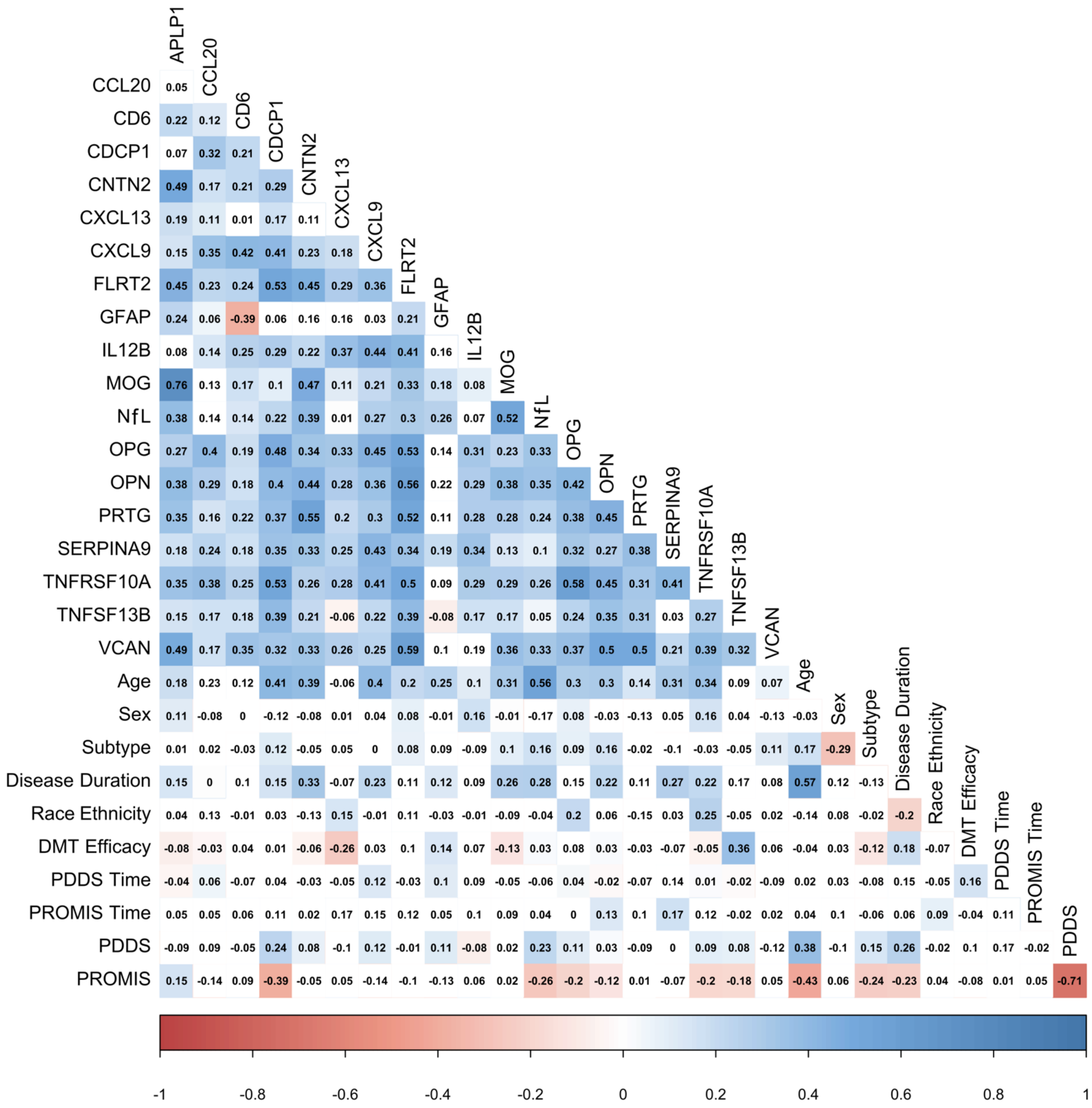

Supplementary Figure 3. Receiver Operating Characteristic (ROC) plots.
For predicting severe versus mild/moderate patient-reported general physical disability using the NIH PROMIS-physical function score (<35 vs. ≥35) in subgroup analyses using the UPMC cohort, we systematically tested multiple machine learning models (LASSO [least absolute shrinkage and selection operator], RF [random forest], XGBoost [Extreme Gradient Boosting] and SVM [support vector machine]). We assessed 95% CIs and p-values for AUC comparisons of all models to the best-performing model nonparametrically by bootstrapping with 1000 replicates. P-values indicate the statistical significance when compared to the best performing model using the combined feature input comprising clinical profile plus serum multi-protein biomarker profile. D-values quantify the difference in performance (AUC) between the two models while accounting for the variability (standard error) of the difference with a higher D-value indicating a greater difference in performance between the models. LASSO: clinical profile (p=.17, D=0.959), serum biomarker profile (p=.03, D=1.901); RF: clinical profile (p=.08, D= 1.399), serum biomarker profile (p=.25 D=0.688); XGBoost: clinical profile (p=.10, D=1.309), serum biomarker profile (p=.01, D=2.317); SVM: clinical profile (p=.08, D=1.411), serum biomarker profile (p=.03, D=2.053).

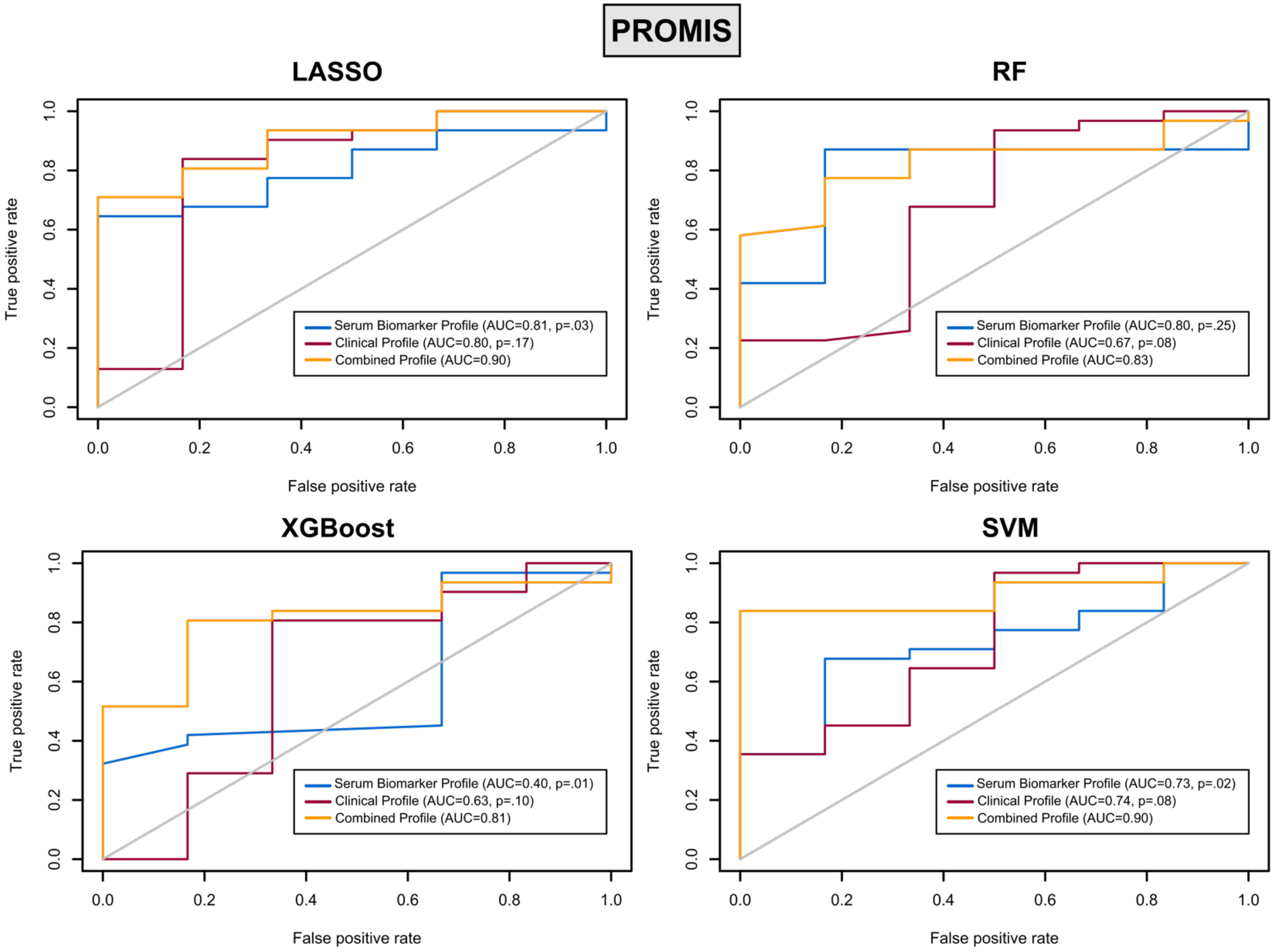

Supplementary Figure 4. Features shared by the best-performing LASSO models.
The Venn diagram shows the overlap among the four best performing LASSO models for predicting the primary endpoint of PDDS and the secondary endpoint of PROMIS-physical function scores. Features in **boldface**: shared by all four LASSO models; Features in *italics*: shared by three LASSO models; Features with underscore: shared by two LASSO models.

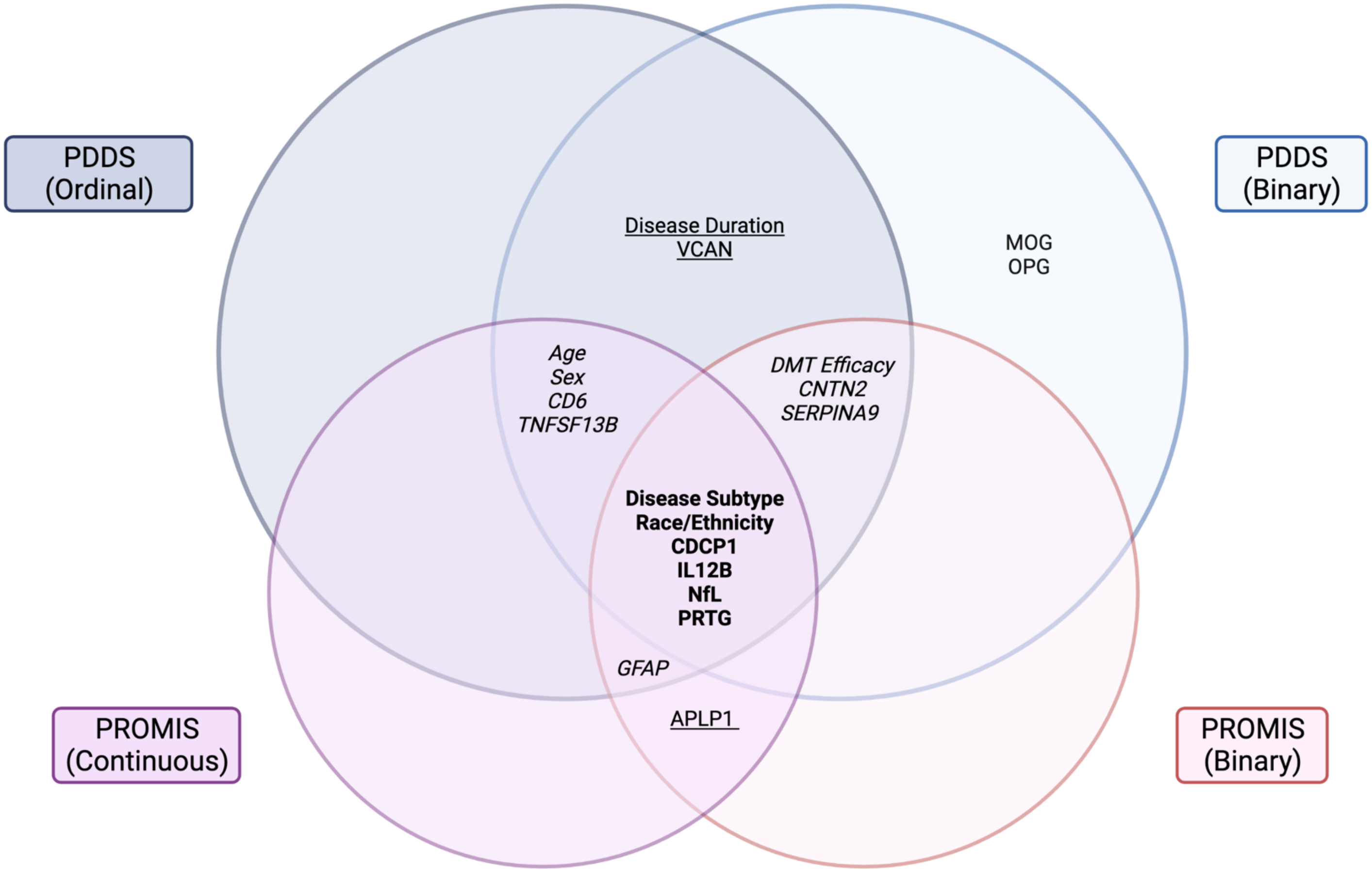

Supplementary Table 1. The 19 proteins in the multi-protein biomarker panel and their designated functional pathways.

Abbreviation	Protein Name	Functional Pathway(s) [1]
**APLP1**	Amyloid beta precursor like protein 1	Myelination, Neuroaxonal Integrity
**CCL20**	Chemokine (C-C motif) ligand 20	Neuroinflammation
**CD6**	Cluster of differentiation 6	Cerebrovascular Function, Immunomodulation, Neuroaxonal Integrity
**CDCP1**	CUB (complement C1r/C1s, Uegf, Bmp1) domain-containing protein 1	Immunomodulation
**CNTN2**	Contactin-2	Neuroaxonal Integrity
**CXCL9**	Chemokine (C-X-C motif) ligand 9,	Immunomodulation, Neuroinflammation
**CXCL13**	Chemokine (C-X-C motif) ligand 13	Immunomodulation, Neuroinflammation
**FLRT2**	Fibronectin leucine-rich transmembrane protein 2	Neuroaxonal Integrity
**GFAP**	Glial fibrillary acidic protein	Cerebrovascular Function, Neuroaxonal Integrity
**IL12B**	Interleukin-12 subunit beta	Immunomodulation, Neuroinflammation
**MOG**	Myelin oligodendrocyte glycoprotein	Myelination
**NfL**	Neurofilament light chain	Neuroaxonal Integrity
**OPG**	Osteoprotegerin	Neuroaxonal Integrity, Neuroinflammation
**OPN**	Osteopontin	Myelination, Neuroaxonal Integrity
**PRTG**	Protogenin	Neuroaxonal Integrity
**SERPINA9**	Serpin family A member 9	Neuroaxonal Integrity
**TNFSF10A**	Tumor necrosis factor ligand superfamily member 10	Neuroaxonal Integrity, Neuroinflammation
**TNFSF13B**	Tumor necrosis factor ligand superfamily member 13B	Immunomodulation, Neuroinflammation
**VCAN**	Versican	Cerebrovascular Function

1. Please refer to the Methods section in the main text and main Figure 2 for functional pathways. A given protein may belong to more than one pathway.

Supplementary Table 2. Feature sets for testing multiple machine learning models.

Machine Learning Models [1]	Feature Sets				
	Clinical Profile	Serum Single Protein	Serum Multi-Protein Profile	Functional Pathways	Combined Profile
**LASSO**	X	X	X		X
**Random Forest**	X		X		X
**Extreme Gradient Boosting**	X		X		X
**Support Vector Machine**	X		X		X
**Stacking Ensemble Learning**					X
**Stacking Classification**				X	X

1. Please refer to the Methods section in the main text for description of the different machine learning models.

Supplementary Table 3. Predictive performance and feature coefficient of benchmark LASSO models using combined clinical profile and a single protein (at a time), clinical profile plus forced single protein of interest (NfL, GFAP) versus clinical profile alone as feature input for predicting severe Patient Determined Disease Steps (PDDS) score (≥4 vs. <4).

Supplementary Table 3a

			Coefficient = 0 [1]						Forced [2]	
	**Clinical Profile**		**CCL20**	**CXCL13**	**OPN**	**NfL**	**GFAP**		**NfL**	**GFAP**
**AUC (95% CI)**	0.85 (0.77, 0.93)		0.86 (0.78, 0.94)	0.85 (0.77, 0.93)	0.86 (0.78, 0.94)	0.86 (0.78, 0.94)	0.85 (0.77, 0.93)		0.85 (0.77, 0.93)	0.85 (0.77, 0.93)
**Sensitivity**	0.60		0.75	0.75	0.75	0.75	0.75		0.60	0.60
**Specificity**	0.81		0.81	0.81	0.81	0.81	0.81		0.81	0.81
**PPV**	0.16		0.16	0.16	0.16	0.16	0.16		0.16	0.16
**NPV**	0.97		0.99	0.99	0.99	0.99	0.99		0.97	0.97
**F1-score**	0.25		0.26	0.26	0.26	0.26	0.26		0.25	0.25
**Protein**	NA		NS	NS	NS	NS	NS		-0.00036	0.00044
**Age**	0.00944		0.00902	0.00926	0.00902	0.01000	0.01000		0.00948	0.00944
**Sex**	-0.07995		-0.06443	-0.07331	-0.06443	-0.07000	-0.07000		-0.08120	-0.08025
**Race / Ethnicity**	0.08149		0.07336	0.07801	0.07336	0.07000	0.08000		0.08214	0.08159
**Disease Subtype**	0.00001		0.00001	0.00001	0.00001	NS	NS		0.00001	0.00001
**Disease Duration**	0.04749		0.01828	0.03499	0.01828	0.02000	0.03000		0.04984	0.04791
**DMT Efficacy**	0.05007		0.03867	0.04519	0.03867	0.04000	0.04000		0.05095	0.05015
**PDDS Time**	0.00033		0.00026	0.00030	0.00026	NS	NS		0.00034	0.00033

Supplementary Table 3b

	Coefficient ≠ 0 [3]						
	**APLP1**	**CD6**	**CDCP1**	**CNTN2**	**CXCL9**	**FLRT2**	**IL12B**
**AUC (95% CI)**	0.85 (0.78, 0.93)	0.86 (0.78, 0.94)	0.85 (0.76, 0.93)	0.85 (0.76, 0.93)	0.84 (0.76, 0.92)	0.85 (0.77, 0.93)	0.85 (0.76, 0.93)
**Sensitivity**	0.80	0.75	0.67	0.75	0.62	0.67	0.67
**Specificity**	0.82	0.81	0.82	0.81	0.83	0.82	0.84
**PPV**	0.21	0.16	0.21	0.16	0.26	0.21	0.32
**NPV**	0.99	0.99	0.97	0.99	0.96	0.97	0.96
**F1-score**	0.33	0.26	0.32	0.26	0.37	0.32	0.43
**Protein**	-0.06045	-0.07983	0.03346	0.02614	0.02399	-0.03496	-0.04675
**Age**	0.00969	0.00931	0.00886	0.00926	0.00926	0.00945	0.00986
**Sex**	-0.07568	-0.09548	-0.07827	-0.08063	-0.08832	-0.07359	-0.07939
**Race/Ethnicity**	0.08656	0.08467	0.07951	0.08049	0.08514	0.08172	0.08716
**Disease Subtype**	0.00001	0.00002	0.00001	0.00001	0.00001	0.00001	0.00001
**Disease Duration**	0.06479	0.06653	0.04719	0.04817	0.06264	0.04335	0.06015
**DMT Efficacy**	0.05184	0.05033	0.05228	0.05210	0.05675	0.04710	0.05904
**PDDS Time**	0.00036	0.00035	0.00033	0.00034	0.00032	0.00032	0.00039
	**Coefficient ≠ 0 [3]**						
	**MOG**	**OPG**	**PRTG**	**SERPINA9**	**TNFRSF10A**	**TNFSF13B**	**VCAN**
**AUC (95% CI)**	0.85 (0.77, 0.93)	0.85 (0.77, 0.93)	0.86 (0.77, 0.94)	0.85 (0.76, 0.93)	0.85 (0.77, 0.93)	0.85 (0.76, 0.93)	0.87 (0.80, 0.95)
**Sensitivity**	0.57	0.80	0.50	0.71	0.75	0.71	0.71
**Specificity**	0.82	0.82	0.81	0.83	0.81	0.83	0.83
**PPV**	0.21	0.21	0.16	0.26	0.16	0.26	0.26
**NPV**	0.96	0.99	0.96	0.97	0.99	0.97	0.97
**F1-score**	0.31	0.33	0.24	0.38	0.26	0.38	0.38
**Protein**	-0.12751	0.01514	-0.08785	-0.02451	-0.05513	0.07100	-0.15700
**Age**	0.01062	0.00927	0.00958	0.00962	0.01003	0.00966	0.00962
**Sex**	-0.07055	-0.07843	-0.08707	-0.07804	-0.07697	-0.08279	-0.08469
**Race/Ethnicity**	0.08758	0.07893	0.08351	0.08196	0.08552	0.07128	0.08795
**Disease Subtype**	0.00001	0.00001	0.00001	0.00001	0.00001	0.00001	0.00001
**Disease Duration**	0.05503	0.03817	0.04663	0.05080	0.06932	0.04979	0.04337
**DMT Efficacy**	0.04890	0.04771	0.04994	0.04812	0.05019	0.04169	0.04720
**PDDS Time**	0.00035	0.00031	0.00034	0.00034	0.00037	0.00032	0.00034

1. Protein biomarkers with coefficients of 0 were not selected (NS) by the individual LASSO models.
2. LASSO models in which either NfL or GFAP were forced into the model.
3. Protein biomarkers with non-zero coefficients were selected by the individual LASSO models (clinical profile and a single protein at a time as feature input).

Abbreviations: AUC = area under the ROC curve, 95% CI = 95% confidence interval, PPV = positive predictive value, NPV = negative predictive value. Please refer to Table 1 and its footnotes in the main text for detailed explanation of the clinical features. Please refer to Figure 2 in the main text and Supplementary Table 1 for the protein biomarkers.

Supplementary Table 4. LASSO model predictive performance and coefficients of the final feature set for predicting PROMIS-physical function scores.

	PROMIS Binary			PROMIS Continuous		
	**Clinical Features Only**	**Multi-Proteins Only**	**Clinical Features + Multi-Proteins**	**Clinical Features Only**	**Multi-Proteins only**	**Clinical Features + Multi-Proteins**
**AUC (95% CI)**	0.80 (0.53, 1.00)	0.81 (0.66, 0.96)	0.90 (0.78, 1.00)			
**Sensitivity**	0.89	0.89	0.93			
**Specificity**	1.00 [X]	0.30	0.57			
**PPV**	1.00 [X]	0.77	0.90			
**NPV**	0.33	0.50	0.67			
**Precision**	1.00 [X]	0.77	0.90			
**F1-score**	0.94	0.83	0.92			
**R^2 (95% CI)**				0.25 (0.06, 0.43)	0.26 (0.16, 0.37)	0.35 (0.29, 0.42)
	**Clinical Features [1]**					
**Age**	-0.00355	-	NS	-0.28044	-	-0.15617
**Sex**	NS	-	NS	NS	-	NS
**Race/Ethnicity**	-0.33248	-	-0.20698	-4.11563	-	-1.04391
**Disease Subtype[1]**	-0.00576	-	-0.00631	NS	-	-0.03201
**Disease Duration**	NS	-	NS	NS	-	NS
**DMT Efficacy[2]**	-0.02264	-	-0.02395	-0.92613	-	NS
**PROMIS Time[3]**	NS	-	NS	0.00082	-	0.00030
	**Protein Features [2]**					
**APLP1**	-	0.22378	0.12854	-	5.61844	5.03634
**CCL20**	-	0.02047	NS	-	0.02698	NS
**CD6**	-	NS	NS	-	1.38090	1.46740
**CDCP1**	-	-0.29921	-0.23801	-	-6.63995	-4.95301
**CNTN2**	-	-0.11284	-0.00068	-	NS	NS
**CXCL13**	-	0.01151	NS	-	NS	NS
**CXCL9**	-	-0.04335	NS	-	NS	NS
**FLRT2**	-	-0.00257	NS	-	NS	NS
**GFAP**	-	-0.04518	-0.01921	-	-0.70370	-0.28375
**IL12B**	-	0.17673	0.11815	-	1.58181	1.16752
**MOG**	-	NS	NS	-	NS	NS
**NfL**	-	-0.17193	-0.09609	-	-5.82478	-2.99074
**OPG**	-	NS	NS	-	-0.82371	-0.40026
**OPN**	-	-0.10643	-0.04977	-	-0.09135	NS
**PRTG**	-	0.27276	0.06075	-	2.95886	1.77987
**SERPINA9**	-	-0.04953	-0.00818	-	NS	NS
**TNFRSF10A**	-	0.11088	NS	-	-1.05103	NS
**TNFSF13B**	-	-0.02354	NS	-	-2.41012	-1.71371
**VCAN**	-	-0.01382	NS	-	3.06650	NS

1. Please refer to Table 1 and its footnotes in the main text for detailed explanation of the clinical features.
2. Please refer to Figure 2 in the main text and Supplementary Table 1 for the protein biomarkers.

[X] The performance of the LASSO model for the binary PROMIS (specificity and PPV) might be influenced by the modest sample size of the test set (n = 20).

Abbreviations: AUC = area under the ROC curve, 95% CI = 95% confidence interval, PPV = positive predictive value, NPV = negative predictive value, PROMIS = Patient-Reported Outcomes Measurement Information System, NS = not selected by LASSO due to zero coefficient.

Supplementary Table 5. Predictive performances of alternative machine learning models in predicting severe patient-reported disability (based on binary PDDS scores [≥4 vs. <4] or binary PROMIS-physical function scores [<35 vs. ≥35]) as compared to the best-performing LASSO models using the combined feature input set.

PDDS	LASSO	Random Forest			XGBoost			Support Vector Machine		
	Clinical Profile + Multi-Protein Profile	Clinical Profile	Multi-Protein Profile	Clinical Profile + Multi-Protein Profile	Clinical Profile	Multi-Protein Profile	Clinical Profile + Multi-Protein Profile	Clinical Profile	Multi-Protein Profile	Clinical Profile + Multi-Protein Profile
**AUC (95% CI)**	0.91 (0.85, 0.97)	0.77 (0.65, 0.89)	0.79 (0.68, 0.89)	0.84 (0.73, 0.94)	0.76 (0.64, 0.89)	0.75 (0.64, 0.87)	0.90 (0.83, 0.97)	0.70 (0.56, 0.84)	0.75 (0.62, 0.89)	0.85 (0.76, 0.95)
**Sensitivity**	0.89	0.50	0.45	0.48	0.58	0.46	0.67	0.60	0.80	0.88
**Specificity**	0.86	0.85	0.86	0.89	0.84	0.83	0.86	0.81	0.82	0.85
**PPV**	0.42	0.42	0.47	0.63	0.37	0.32	0.42	0.16	0.21	0.37
**NPV**	0.99	0.89	0.84	0.81	0.93	0.90	0.94	0.97	0.99	0.99
**F1-score**	0.57	0.46	0.46	0.55	0.45	0.37	0.52	0.25	0.33	0.52
**PROMIS**	**LASSO**	**Random Forest**			**XGBoost**			**Support Vector Machine**		
	**Clinical Profile + Multi-Protein Profile**	**Clinical Profile**	**Multi-Protein Profile**	**Clinical Profile + Multi-Protein Profile**	**Clinical Profile**	**Multi-Protein Profile**	**Clinical Profile + Multi-Protein Profile**	**Clinical Profile**	**Multi-Protein Profile**	**Clinical Profile + Multi-Protein Profile**
**AUC (95% CI)**	0.90 (0.78, 1.00)	0.67 (0.38, 0.97)	0.80 (0.61, 0.98)	0.83 (0.68, 0.87)	0.63 (0.31, 0.96)	0.40 (0.14, 0.66)	0.81 (0.65, 0.97)	0.74 (0.49, 0.98)	0.73 (0.52, 0.93)	0.90 (0.80, 1.00)
**Sensitivity**	0.93	0.90	0.87	0.90	0.86	0.84	0.90	0.91	0.86	0.86
**Specificity**	0.57	0.43	0.33	0.43	0.25	0.17	0.38	0.75	0.50	0.50
**PPV**	0.90	0.87	0.87	0.87	0.81	0.68	0.84	0.97	0.97	0.97
**NPV**	0.67	0.50	0.33	0.50	0.33	0.33	0.50	0.50	0.17	0.17
**F1-score**	0.92	0.89	0.87	0.89	0.83	0.75	0.87	0.94	0.91	0.91

Abbreviations: AUC = area under the ROC curve, 95% CI = 95% confidence interval, PPV = positive predictive value, NPV = negative predictive value

Supplementary Table 6. Predictive performances across different *stacking ensemble* models in predicting severe patient-reported disability (based on binary PDDS scores [≥4 vs. <4] or binary PROMIS-physical function scores [<35 vs. ≥35]).

LASSO [1]	-	-		Random Forest [2]	-	-
	**PDDS**	**PROMIS**			**PDDS**	**PROMIS**
**AUC (95% CI)**	0.89 (0.82, 0.97)	0.80 (0.63, 0.97)		**AUC (95% CI)**	0.84 (0.74, 0.95)	0.86 (0.74, 0.98)
**Sensitivity**	0.67	0.93		**Sensitivity**	0.70	0.90
**Specificity**	0.86	0.44		**Specificity**	0.85	0.38
**PPV**	0.42	0.84		**PPV**	0.37	0.84
**NPV**	0.94	0.67		**NPV**	0.96	0.50
**F1-Score**	0.52	0.88		**F1-Score**	0.48	0.87

XGBoost [3]	-	-		Support Vector Machine [4]	-	-
	**PDDS**	**PROMIS**			**PDDS**	**PROMIS**
**AUC (95% CI)**	0.82 (0.72, 0.91)	0.67 (0.44, 0.90)		**AUC (95% CI)**	0.68 (0.56, 0.79)	0.69 (0.46, 0.91)
**Sensitivity**	0.75	0.90		**Sensitivity**	0.90	0.90
**Specificity**	0.84	0.38		**Specificity**	0.43	0.43
**PPV**	0.32	0.84		**PPV**	0.87	0.87
**NPV**	0.97	0.50		**NPV**	0.50	0.50
**F1-Score**	0.44	0.87		**F1-Score**	0.89	0.89

1. Stacking ensemble learning using LASSO as the Level 2 model (Refer to Methods in the main text and Figure 3)
2. Stacking ensemble learning using Random Forest as the Level 2 model
3. Stacking ensemble learning using XGBoost as the Level 2 model
4. Stacking ensemble learning using SVM as the Level 2 model

Abbreviations: AUC = area under the ROC curve, 95% CI = 95% confidence interval, PPV = positive predictive value, NPV = negative predictive value
